# Serum Uric Acid Levels Predict Mortality Risk in Male Amyotrophic Lateral Sclerosis Patients

**DOI:** 10.3389/fneur.2021.602663

**Published:** 2021-03-11

**Authors:** Liu-Qing Xu, Wei Hu, Qi-Fu Guo, Guo-Rong Xu, Ning Wang, Qi-Jie Zhang

**Affiliations:** ^1^Department of Neurology, Fujian Institute of Neurology, The First Affiliated Hospital, Fujian Medical University, Fuzhou, China; ^2^Fujian Key Laboratory of Molecular Neurology, Fujian Medical University, Fuzhou, China

**Keywords:** amyotrophic lateral sclerosis, uric acid, prognosis, survival, biomarker

## Abstract

**Objective:** To explore the associations between serum uric acid levels with survival in male and female ALS patients.

**Methods:** A longitudinal cohort study was carried out including 313 sporadic and 16 familial ALS patients with repeated serum uric acid measurements. Multivariate Cox regression models were used to evaluate the survival-related factors.

**Results:** There were 207 male and 122 female, and the mean age of onset was 55.7 ± 11.2 years old. The male patients had significantly higher baseline uric acid levels than that in female patients (342.4 ± 91.4 vs. 279.3 ± 71.4 μmol/L; *p* < 0.0001). The uric acid levels were inversely associated with the decline rate of ALSFRS-R per month (ΔALSFRS-R). After multivariate Cox regression analysis, a survival advantage was found in male, but not female, with higher serum uric acid levels. In males, a shorter diagnostic delay (≤10 m), lower BMI at baseline (≤18.70 kg/m^2^), faster disease progression (ΔALSFRS-R > 0.63), and lower baseline uric acid levels (≤292 μmol/L, HR: 1.936; 95% CI: 1.334–2.810) were associated with a shorter survival. During follow-up, the serum uric acid levels were not significantly altered over time.

**Conclusion:** There is an inverse correlation between baseline serum uric acid levels and risk of death, prominently in male ALS patients.

## Introduction

Amyotrophic lateral sclerosis (ALS) is a chronic and incurable adult-onset neurodegenerative disorder that primarily impairs upper and lower motor neurons (1). Clinically, it is characterized by male predominance, upper limb onset, relentlessly progressive muscle weakness and atrophy, and a variable natural history (2). Until now, no efficacious treatment is available, and a majority of ALS patients ultimately die from respiratory muscle paralysis, typically 2–5 years after symptom onset. The etiology of ALS remains unclear. A total of 90–95% of ALS cases have no family history of the disease and are of sporadic nature, whereas only 5–10% are familial with positive family history of gene mutations (3). Pathophysiological mechanisms that contribute to the development and progression of ALS are complicated, including dysfunction of RNA metabolism, impaired proteostasis, impaired DNA repair, mitochondrial dysfunction and oxidative stress, axonal transport defects, glutamate excitotoxicity, and neuroinflammation (4). Increasing evidence supports that oxidative stress may play a critical role in the pathogenesis of ALS, the SOD1 protein is an important anti-oxidative enzyme in cytoplasm, and the ALS mutant TDP-43 and FUS cytoplasmic inclusions also co-localize with stress granule *in vitro* (5, 6). Based on the hypothesis of oxidative stress, edaravone, a free-radical scavenger and antioxidant that alleviates the oxidative stress on the nerves and slows the disease progression, has been approved to be an effective disease-modifying treatment for ALS (7).

Recently, serum uric acid is gaining increasing attention as a candidate indicator of risk and progression in ALS (8, 9). Uric acid, one of the metabolic products of purine, is a naturally occurring antioxidant. In clinical, the edaravone-treated ALS patients showed significantly increased blood uric acid levels as compared to the untreated cases (10). Previous studies from different research groups showed consistently that ALS cases had significantly lower blood uric acid concentrations than age- and gender- matched healthy controls (11–15). However, the association between blood uric acid levels and survival or progression of ALS was controversial. Zheng et al. haven't observed a link between serum uric acid level and survival among the sporadic ALS patients in Chinese population (16). In our previous study, we compared the routinely laboratory measurements between the ALS patients with short-duration (≤3 years) and long-duration (>3 years) group, which indicated that patients with increased uric acid levels showed a prolonged survival (17). After plasma component analysis using surface enhanced Raman spectroscopy, we further found that the ALS patients with short duration showed a significantly decreased antioxidant level, and the metabolism of glutathione was correlated with the disease progression (17). In the current study, we carried out a prospective cohort study including 329 ALS patients from Southeast of China, aiming to explore the associations between serum uric acid levels and survival.

## Materials and Methods

### Patients

The ALS patients were enrolled serially from the department of neurology, first affiliated hospital of Fujian medical university from December 2014 to September 2019. All the patients were diagnosed with definite, probable, or possible ALS on the basis of the revised ALS Escorial criteria, and the diagnosis was confirmed by two professional neurologists (18). Patients with serum uric acid levels more than 420 μmol/L were defined as hyperuricemia according to the Chinese multi-disciplinary consensus on the diagnosis of hyperuricemia (19). Patients who suffered from renal diseases, malignant tumors and other diseases that might affect serum uric acid levels and survival were excluded. Patients who refused to undergo serum uric acid testing were also excluded. This study was approved by the Ethics Board of the First Affiliated Hospital of Fujian Medical University. Written informed consent was obtained from each participant.

### Clinical Data Collection and Serum Uric Acid Measurements

Clinical profiles were collected detailed at the time of diagnosis (baseline), including gender, height, weight, age of onset, site of first symptom onset, family history of ALS, medical history (especially the renal diseases and malignant tumors), neurophysiological tests (EMG), and treatments. Diagnostic delay was defined as the time interval in months from symptom onset to an identified diagnosis of ALS. The Functional Rating Scale-Revised (ALSFRS-R, range from 1 to 48 score) was used to assess the level of neurological impairment. The disease progression at baseline was assessed by the linear change rate of ALSFRS-R (ΔALSFRS-R), which was calculated by the equation: ΔALSFRS-R = (48 – ALSFRS-R score at baseline)/duration in months from symptom onset to baseline clinical assessment. All the recruited patients were followed up regularly (every 3–6 months) in person or by telephone (for those patients at the later disease stage with mobility inconvenience). A treatment of riluzole with 50 mg, twice per day for longer than 3 months was defined as “use of riluzole.” An acceptance of noninvasive positive pressure ventilation (NIPPV) for longer than 4 h per day was defined as “use of NIPPV.” The ALS staging was defined based on the King's College staging system (20). For survival analysis, all the cases were followed up at least more than 12 months (the last visit was April 2020). The primary outcome was defined by either tracheotomy or death. The blood sample was drawn from the peripheral vein after 8 h of fasting, and the serum was separated out to quantify the uric acid level in the department of laboratory, first affiliated hospital of Fujian medical university. For the patients whose serum uric acid levels were quantified in other medical centers with certified labs, we recorded the uric acid results that were performed within 3 month before the initial clinical assessment. The reported upper limit of normal uric acid ranged from 416 to 440 μmol/L for male, and 339 to 430 μmol/L for female.

### Statistical Analysis

The statistical analysis was performed by the SPSS statistical software version 17.0 for Windows (SPSS, Inc, IL, USA). The categorical variables were expressed as percentage, including gender, family history, site of onset, diagnostic categories, use of riluzole, acceptance of PEG, and use of NIPPV. For the continuous variables with normal distribution, they were expressed as mean and standard deviation (SD), and for those variables without normal distribution, they were expressed as median and interquartile range (IQR). Student's *T*-test was applied to analyze the difference in the serum uric acid levels between the male and female patients, bulbar onset and spinal onset patients. One-way ANOVA was employed to compare the uric acid levels in ALS patients with different stages. The cut-off values for age of onset, diagnostic delay, body mass index, ALSFRS-R score, ΔALSFRS-R, and serum uric acid level were identified using X-tile software (non-commercial, version 3.6.1, Yale University, USA). The survival analysis was performed by Kaplan Meier analysis (Log rank test), and adjusted using Multivariate Cox regression analysis (Backward Stepwise, Wald).

The survival-related factors for Multivariate Cox regression analysis were chose according to previous studies (21). Correlations were analyzed with the Pearson coefficient (*r*) or Spearman coefficient (*r*_s_). *P*-value <0.05 was considered statistically significant.

## Results

### Clinical Features of ALS Patients

A total of 346 ALS patients with serum uric acid testing were enrolled serially. Among them, 1 case combined with chronic reduction of renal function, and 8 cases combined with cancers (1 with hepatic carcinoma, 1 with nasopharyngeal carcinoma, 1 with glioma, 2 with breast cancer, 2 with lung cancer, and 1 with rectal cancer). Eight patients were lost to follow-up. The remaining 329 cases were included into the final survival analysis. There were 207 male and 122 female (ratio of male to female was 1.7:1). Sixteen patients had a positive family history of ALS, and 313 cases were sporadic. The mean age of first symptom onset was 55.7 ± 11.2 years old. According to the site of onset, 71 cases were bulbar-onset, 165 cases were upper limb-onset, and 93 cases were lower limb-onset. The median diagnostic delay was 11.0 months (IQR: 6.0, 16.0). The median ALSFRS-R score at baseline was 41 (IQR: 38, 44). The median serum uric acid level at baseline was 308 μmol/L (IQR: 256–376), and 51 patients were combined with hyperuricemia without symptoms of gout. At the last follow-up visit (April, 2020), there were 94 (28.6%) patients still alive, and the remaining 235 (71.4%) patients reached the combined endpoint, of which 218 cases died and 17 cases underwent tracheotomy. The detailed demographic and clinical features were showed in Table 1.

**Table 1:** Clinical characteristic of patients with ALS.

**variables and subgroups**	**No. (%), mean ± SD or median (IQR)**
**Gender**	
Male	207 (62.9%)
Female	122 (37.1%)
**Family history**	
Yes	16 (4.9%)
No	313 (95.1%)
Age of onset, mean ± SD, y	55.7 ± 11.2
**Site of onset**	
Bulbar	71(21.6%)
Upper limb	165 (50.1%)
Lower limb	93 (28.3%)
**Diagnostic categories**	
Definite	193 (58.7%)
Probable	92 (28.0%)
Possible	44 (13.3%)
Diagnostic delay, median (IQR), mo	*n* = 329, 11.0 (6.0, 16.0)
BMI at baseline, mean ± SD, kg/m^2^	*n* = 329, 21.50 ± 2.83
ALSFRS-R score at baseline, median (IQR)	*n* = 329, 41(38, 44)
ΔALSFRS-R, median (IQR)	*n* = 329, 0.67(0.36, 1.20)
**Use of riluzole**	
Yes	233 (70.8%)
No	96 (29.2%)
**Acceptance of PEG**	
Yes	29 (8.8%)
No	300 (91.2%)
**Use of NIPPV**	
Yes	52 (15.8%)
No	277 (84.2%)
**Primary outcome**	
Death/tracheotomy	235 (71.4%)
Alive	94 (28.6%)
Serum uric acid at baseline, median (IQR), μmol/L	*n* = 329, 308 (256, 376)

*ALS, amyotrophic lateral sclerosis; ALSFRS-R, Amyotrophic Lateral Sclerosis Functional Rating Scale–Revised; BMI, body mass index; PEG, percutaneous endoscopic gastrostomy; NIPPV, non-invasive positive pressure ventilation; IQR, Interquartile range; SD, Standard deviation*.

### Increased Serum Uric Acid Levels Were Linked With Lower Mortality Risk

**Figure 1:**
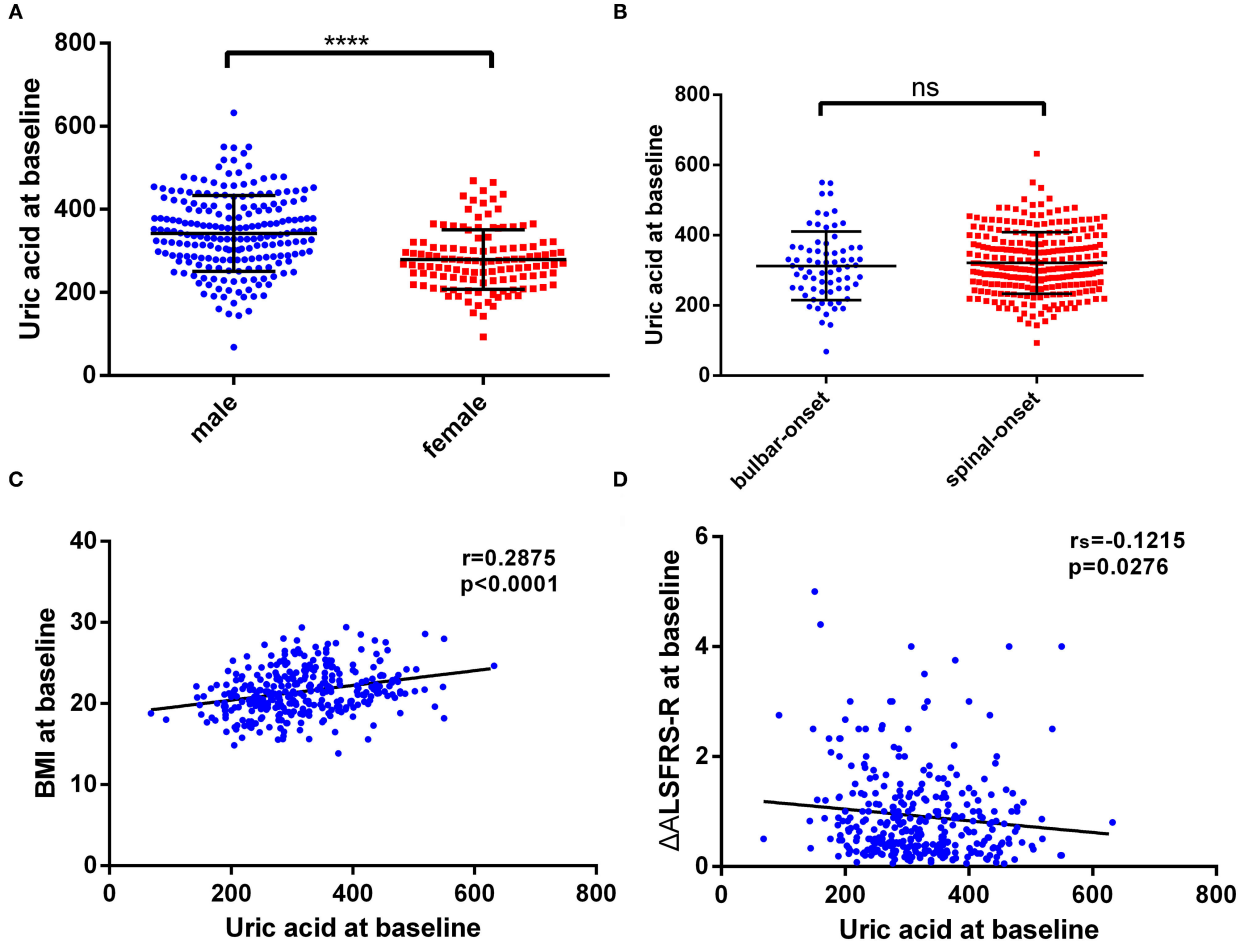
The distribution of baseline serum uric acid and its correlation with BMI and disease progression rate. **(A)** The distribution of uric acid levels in male and female patients. **(B)** The distribution of uric acid levels in bulbar-onset and spinal-onset patients. **(C)** The correlation between uric acid levels and BMI. **(D)** The correlation between uric acid levels and ΔALSFRS-R. The distribution of uric acid was expressed as the mean with standard deviation. ****P < 0.0001.

Firstly, we performed the distribution analysis of serum uric acid levels among male and female patients, and the result showed that the male ALS cases had significantly higher uric acid concentrations than that in female cases (342.4 ± 91.4 vs. 279.3 ± 71.4 μmol/L; *t* = 6.546, *p* < 0.0001; Figure 1A). However, serum uric acid levels were not significantly different between the bulbar onset and spinal onset patients (312.6 ± 97.7 vs. 320.8 ± 87.6 μmol/L; *t* = 0.6790, *p* = 0.4976; Figure 1B). Additionally, serum uric acid levels at baseline were positively associated with BMI (*r* = 0.2875, *p* < 0.0001; Figure 1C), and inversely associated with disease progression rate (ΔALSFRS-R, *r*_s_ = −0.1215, *p* = 0.0276; Figure 1D); whereas no significant association was found between serum uric acid levels and ALSFRS-R scores (*r*_s_ = 0.02669, *p* = 0.6295).

**Figure 2:**
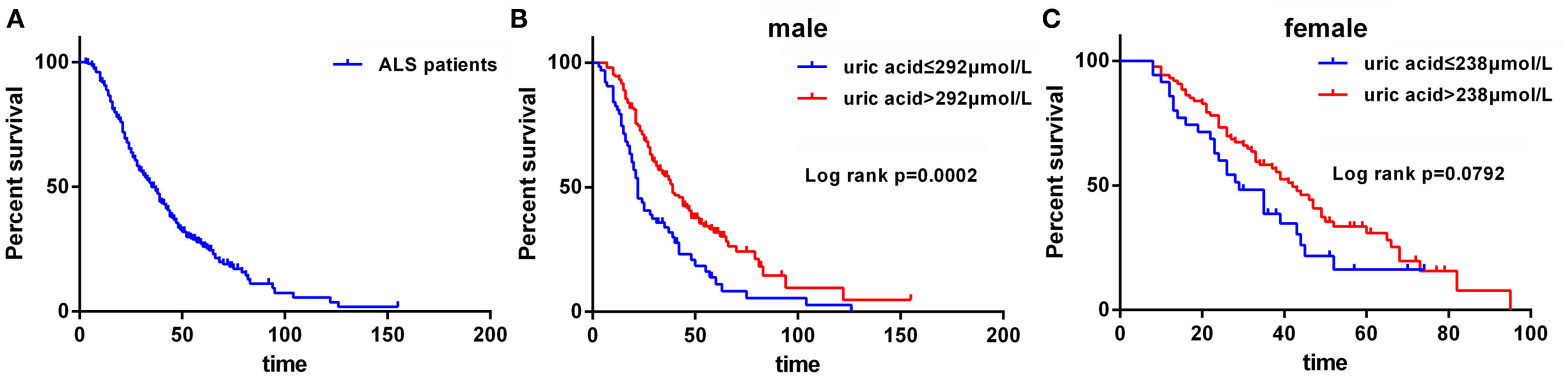
The survival analysis for ALS patients. **(A)** The survival curve for all ALS patients. **(B)** The survival curves for male patients with uric acid ≤ 292 μmol/L vs. uric acid > 292 μmol/L. **(C)** The survival curves for female patients with uric acid ≤ 238 μmol/L vs. uric acid > 238 μmol/L.

Secondly, in light of the fact that levels of uric acid were significantly different in male and female ALS patients, we performed the survival analysis based on the subgroup of gender. The cut-off values of age of onset, BMI at baseline, diagnostic delay, ALSFRS-R, ΔALSFRS-R, uric acid level for male, and uric acid level for female were 55 years old, 18.70 kg/m^2^, 10 months, 43 score, 0.63, 292 μmol/L, and 238 μmol/L, respectively, which were identified using X-tile software. The median survival time for all ALS patients was 35 months (Figure 2A). The median survival time for male and female patients was 34.0 and 39.0 months, respectively (*p* = 0.6322). The median survival for patients with bulbar-onset and spinal-onset ALS was 27.0 and 37.0 months, respectively (*p* = 0.0545). Among the male ALS patients, lower serum uric acid levels (≤292 μmol/L) were significantly linked with shorter survival using Kaplan Meier analysis (Figure 2B). Among the female ALS patients, the median survival for high serum uric acid group (>238 μmol/L) and low serum uric acid group (≤238 μmol/L) was 42 and 29 months (*p* = 0.0792) (Figure 2C).

Thirdly, multivariate Cox regression model was used to further explore the survival-related factors after adjustment for variables, including age of onset, site of onset, BMI at baseline, ALSFRS-R score at baseline, progression rate (ΔALSFRS-R), diagnostic delay, use of riluzole, use of NIPPV, acceptance of PEG, and serum uric acid levels. Among the male group, a shorter diagnostic delay (≤10 m, HR: 2.353; 95% CI: 1.616–3.428), lower BMI at baseline (≤18.70 kg/m^2^, HR: 2.619; 95% CI: 1.666–4.116), without use of NIPPV (HR: 1.541; 95% CI: 1.006–2.360), faster disease progression (ΔALSFRS-R > 0.63, HR: 4.208; 95% CI: 2.799–6.326), and lower uric acid levels at baseline (≤292 μmol/L, HR: 1.936; 95% CI: 1.334–2.810) were associated with a shorter survival (Table 2). Among the female group, an older age of onset (>55 y, HR: 2.096; 95% CI: 1.294–3.395), shorter diagnostic delay (≤10 m, HR: 2.393; 95% CI: 1.488–3.848), lower BMI at baseline (≤18.70 kg/m^2^, HR: 2.381; 95% CI: 1.279–4.431), and faster disease progression (ΔALSFRS-R > 0.63, HR: 3.351; 95% CI: 1.986–5.655) were associated with a higher mortality risk (Table 2).

**Table 2:** Multivariable model in male and female using multivariate Cox survival analysis (backward stepwise, Wald).

**variables and subgroups**	**β**	**SE**	**Wald**	***p*-value**	**HR (95%CI)**
**Male**					
**diagnostic delay**					
>10 m					1.0 (Reference)
≤10 m	0.856	0.192	19.885	<0.001	2.353 (1.616, 3.428)
**BMI at baseline**					
>18.70					1.0 (Reference)
≤18.70	0.963	0.231	17.396	<0.001	2.619 (1.666, 4.116)
**use of NIPPV**					
Yes					1.0 (Reference)
No	0.432	0.217	3.955	0.047	1.541 (1.006, 2.360)
**ΔALSFRS-R**					
≤0.63					1.0 (Reference)
>0.63	1.437	0.208	47.688	<0.001	4.208 (2.799, 6.326)
**Uric acid at baseline**					
>292 μmol/L					1.0 (Reference)
≤292 μmol/L	0.661	0.19	12.098	0.001	1.936 (1.334, 2.810)
**Female**					
**age of onset**					
≤55 y					1.0 (Reference)
>55 y	0.74	0.246	9.05	0.003	2.096 (1.294, 3.395)
**diagnostic delay**					
>10 m					1.0 (Reference)
≤10 m	0.873	0.242	12.963	<0.001	2.393 (1.488, 3.848)
**BMI at baseline**					
>18.70					1.0 (Reference)
≤18.70	0.867	0.317	7.486	0.006	2.381 (1.279, 4.431)
**ΔALSFRS-R**					
≤0.63					1.0 (Reference)
>0.63	1.209	0.267	20.529	<0.001	3.351 (1.986, 5.655)

*ALSFRS-R, Amyotrophic Lateral Sclerosis Functional Rating Scale–Revised; BMI, body mass index; NIPPV, non-invasive positive pressure ventilation; HR, hazard ratio; CI, confidence interval*.

**Figure 3:**
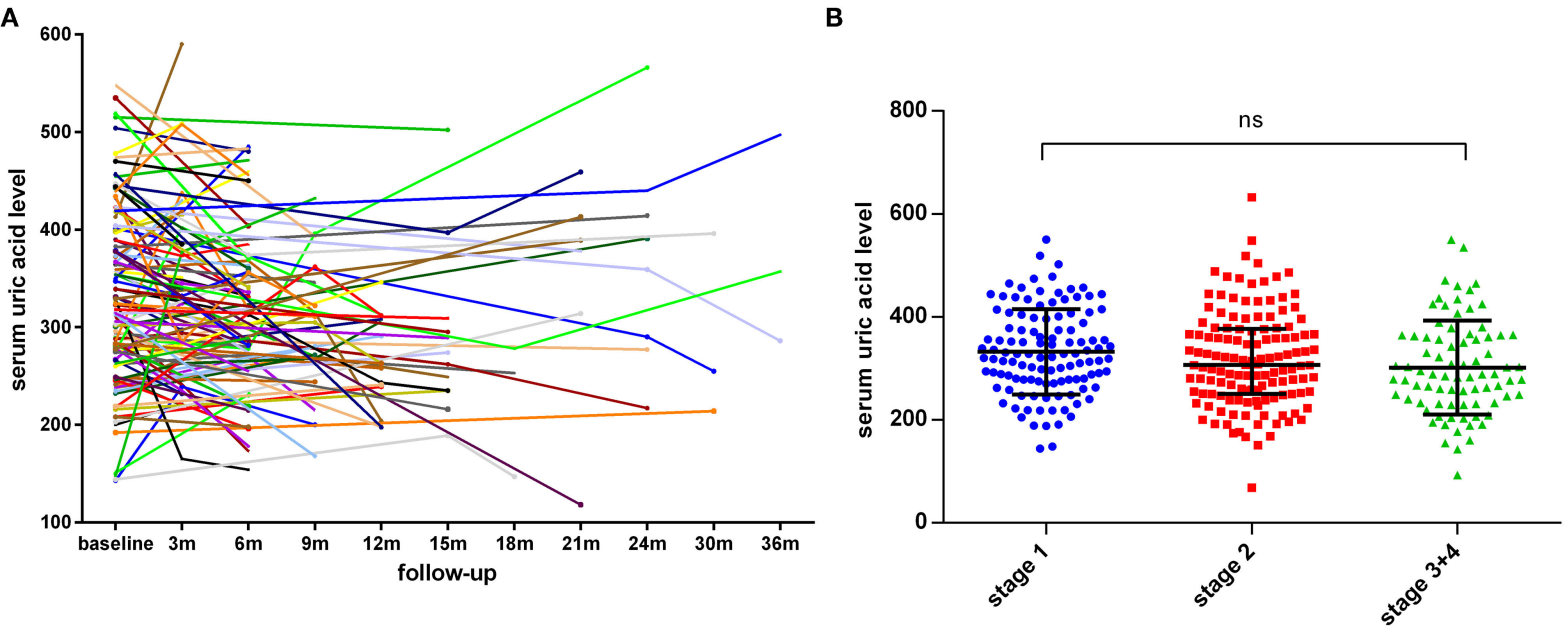
The distribution of uric acid levels at different time points of follow-up and different King's College stages. **(A)** The line chart of uric acid levels for 112 patients at different time points of follow-up. **(B)** The serum uric levels in ALS patients with different King's College stages. The median concentrations in stage 1, stage 2, and stge 3 + 4 were 328, 307, and 288 μmol/L, respectively, (*p* = 0.0622). Serum uric acid levels were expressed as median with interquartile range.

Finally, we recorded 146 repeated serum uric acid measurements for 112 patients at different time points of follow-up, and the distribution of serum uric acid levels at baseline and at different time points of visit was shown in Figure 3A, which indicated that the concentrations of serum uric acid were highly variable over time. Additionally, we assessed the ALS stage according to the King's College staging system, and the median concentrations of uric acid in stage 1, stage 2, and stge 3 + 4 were 328, 307, and 288 μmol/L, respectively (*p* = 0.0622, Figure 3B).

## Discussion

ALS is a progressive motor neuron degenerative disease with highly variable clinical course, and the time from disease onset to death ranges from 6 months to 50 years (22). For the time being, the causative pathogenic mechanisms in ALS remain under investigated, and it still lacks a reliable biomarker, especially in sporadic cases. There is some evidence that oxidative stress may be the primary initiating factor in ALS pathogenesis (23). O'Reilly et al. carried out a prospective study with 319,617 individuals, detected the pre-diagnostic uric acid levels, and found an inverse association between uric acid concentrations and the risk of ALS (24). However, the association of uric acid with the future risk of ALS was not noted in the Swedish Apolipoprotein-Related Mortality Risk study (25). Compared to the healthy controls, ALS patients have significantly lower serum uric acid levels, which have been confirmed by diverse case-control studies from Italian, Japanese, Korean, and Chinese ALS patients (11–15). Collectively, as an important antioxidant in human beings, uric acid appears to play a protective role in the development of ALS.

Subsequently, the prognostic value of serum uric acid in disease severity, progression, and survival in ALS patients warrants further investigation. In the present study, we observed that the male patients had a higher level of uric acid at baseline than that in female patients. The concentrations of uric acid were positively correlated with BMI, whereas not with site of onset and ALSFRS-R score. A significantly inverse association was found between uric acid levels and disease progression rate (expressed as ΔALSFRS-R), which was consistent with previous studies (12, 13). After multivariate Cox regression survival analysis, baseline serum uric acid level was an independent predictive factor for survival among male ALS patients, even after multiple adjustments of age of onset, site of onset, BMI at baseline, ALSFRS-R score at baseline, progression rate (ΔALSFRS-R), diagnostic delay, and use of riluzole. In male patients, the median survival for high serum uric acid group (>292 μmol/L) and low serum uric acid group (≤292 μmol/L) was 39 and 22 months. In female patients, lower uric acid levels (≤238 μmol/L) also showed a shorter survival than higher group (>238 μmol/L), and the median survival time was 29 and 42 months, respectively, while without statistically significant difference. Previously, Paganoni et al. also reported a dose-dependent effect of serum uric acid in survival among the male ALS patients, and they indicated that an increase of 1 mg/dl of uric acid levels could lead to a 39% reduction in risk of death (26). O'Reilly et al. observed a significant association between the uric acid levels and outcomes in males, whereas the survival advantage was attenuated after adjustment for BMI (27). Taken together, it seemed that there was a survival advantage in male, but not female, with higher serum uric acid levels at baseline. However, the median survival time for male and female patients was 34.0 and 39.0 months, respectively, and the male patients haven't shown a better prognosis than females. The underlying mechanisms are still largely unexplained, and there may be other disease modifying factors.

In the present study, we also compared the concentrations of uric acid during different time points of follow-up for 112 patients, and the uric acid levels were highly variable over time. Previously, Chen et al. performed a longitudinal observation in 81 ALS patients, and found that uric acid levels were significantly decreased in the second hematological examinations (15). Present evidence also supports that the initiation of treatments or diet/lifestyle change after diagnosis of ALS might influence the metabolism of UA. Nagase et al. observed that the plasma uric acid levels were significantly increased after edaravone treatment (10). In this study, we also observed relatively lower serum uric acid levels at later disease stages (stage 1 vs. stage 2 vs. stage 3 + 4), although not statistically significant. Besides, the serum uric acid levels might also be decreased in ALS patients with bulbar involvement. In future studies, it will be useful to design longitudinal cohort study with regular time interval of disease severity assessment and serum uric acid measurements.

There were some limitations in this study. Although a shorter median survival time was observed in female patients with lower uric acid levels, no significant difference was obtained statistically, which may partly due to a small sample size of female cases. Additionally, some other prognostic factors for survival were not included and analyzed, for instance, pulmonary function parameters (FVC).

In conclusion, the male ALS patients have significantly higher levels of serum uric acid than female patients. There is an inverse correlation between baseline serum uric acid levels and risk of death, prominently in male ALS patients.

## Data Availability Statement

The raw data supporting the conclusions of this article will be made available by the authors, without undue reservation.

## Ethics Statement

The studies involving human participants were reviewed and approved by the Ethics Board of the First Affiliated Hospital of Fujian Medical University. The patients/participants provided their written informed consent to participate in this study.

## Author Contributions

NW and Q-JZ: study concept and design, critical revision of the manuscript for important intellectual content, obtaining of funding and study supervision. L-QX, WH, Q-FG, G-RX, and Q-JZ: acquisition and interpretation of data. Q-JZ, L-QX, and WH: drafting of the manuscript. L-QX and WH contributed equally to this work. All authors read and approved the final manuscript.

## Conflict of Interest

The authors declare that the research was conducted in the absence of any commercial or financial relationships that could be construed as a potential conflict of interest.
